# Enhancing Hydrogen Sulfide Detection at Room Temperature Using ZIF-67-Chitosan Membrane

**DOI:** 10.3390/membranes13030333

**Published:** 2023-03-14

**Authors:** Ashraf Ali, Ahmed Alzamly, Yaser E. Greish, Reem H. Alzard, Hesham F. El-Maghraby, Naser Qamhieh, Saleh T. Mahmoud

**Affiliations:** 1 Department of Physics, United Arab Emirates University, Al-Ain 15551, United Arab Emirates; 2 Department of Chemistry, United Arab Emirates University, Al-Ain 15551, United Arab Emirates; 3 Department of Ceramics, National Research Centre, Cairo 68824, Egypt

**Keywords:** zeolitic imidazole frameworks, gas sensors, H_2_S gas, chitosan

## Abstract

Developing new materials for energy and environment-related applications is a critical research field. In this context, organic and metal–organic framework (MOF) materials are a promising solution for sensing hazardous gases and saving energy. Herein, a flexible membrane of the zeolitic imidazole framework (ZIF-67) mixed with a conductivity-controlled chitosan polymer was fabricated for detecting hydrogen sulfide (H_2_S) gas at room temperature (RT). The developed sensing device remarkably enhances the detection signal of 15 ppm of H_2_S gas at RT (23 °C). The response recorded is significantly higher than previously reported values. The optimization of the membrane doping percentage achieved exemplary results with respect to long-term stability, repeatability, and selectivity of the target gas among an array of several gases. The fabricated gas sensor has a fast response and a recovery time of 39 s and 142 s, respectively, for 15 ppm of H_2_S gas at RT. While the developed sensing device operates at RT and uses low bias voltage (0.5 V), the requirement for an additional heating element has been eliminated and the necessity for external energy is minimized. These novel features of the developed sensing device could be utilized for the real-time detection of harmful gases for a healthy and clean environment.

## 1. Introduction

Human health has been, and always will be, under threat because of human activities. The exhaust from industries as well as the vehicles and byproducts emanating from various processes in refineries and other industries have made the environment more hostile every day. The presence of harmful and toxic gases in the environment must be constantly monitored to avoid any unforeseen adverse effects. Therefore, materials that can be used to detect these harmful gases in the atmosphere from among the non-harmful ones play a considerably important role.

Hydrogen sulfide (H_2_S) is one of the most hazardous gases originating from industrial processes involving crude petroleum, natural gas, and landfills [1,2,3,4]. H_2_S also evolves from wastewater treatment, tanneries, glue and dye production, and drilling and mining industries [4,5,6,7]. Even at low concentrations, exposure to H_2_S can cause many adverse outcomes, ranging from loss of consciousness to certain death. Thus, the threat posed by H_2_S not only requires real-time monitoring but also calls for immediate presentment, which would signify the difference between life and death in these situations.

Materials that exhibit changes in their physical and or optical properties are critical candidates for sensing materials in gas-sensing devices [8,9]. Ideal candidates have properties that enhance the interaction between the sensing material and the target gas molecules. One of these properties that have proven advantageous is material porosity. Materials such as metal–organic frameworks (MOFs) synthesized by linking metal cations or metal clusters with organic linkers possess this desirable porosity [10,11]. Another class of material that originates from the same family are zeolitic imidazole frameworks (ZIFs), which comprise inorganic metal cations (M^2+^) and imidazolate-type ligands [12,13]. These materials exhibit promising features, such as chemical resistance and very large surface areas [13]. Currently, more than 150 novel imidazolate MOFs structures have been synthesized [13,14]. The literature demonstrates that among the available ZIFs, ZIF-67 and ZIF-8 have been used as sensing materials for gas-sensing applications [15,16,17]. The structural properties that complement the task at hand are extreme stability with a very high surface area and easy synthesis [12,18,19,20]. In comparison with conventional sensing materials, such as conducting polymers and semiconductor metal oxides, ZIF-based structures provide advantages, such as high sensitivity, selectivity, and stability [21,22,23,24,25].

The chitosan (CS) polymer, along with ionic liquid (IL) glycerol, reportedly detects H_2_S gas at 15 ppm at an operating temperature of 80 °C [26]. Meanwhile, the literature demonstrates that the density of ZIF structures affects the number of active sites that facilitate the detection of H_2_S at room temperature (RT) [10]. The incorporation of IL into the matrix enhances the conductivity of the sensing material in detecting the target gas. Conventionally, ZIF-67 has not been used to detect H_2_S gas [13,27,28] but rather other VOC gases [27,28,29,30,31,32] and inorganic gases [33,34,35]. It has also been used in water purification [15,36,37,38,39] CO_2_ detection and separation [14,40,41,42,43], organic dyes [19,36,44], electrochemical sensors [16,45,46], and energy applications [23,47,48]. To the best of our knowledge, ZIF-67 has not been reported in combination with CS for H_2_S gas detection applications.

The main objectives of this study are to enhance the detection of H_2_S gas in the CS–IL matrix by doping it with varying concentrations of ZIF-67 in terms of weight% (wt%) and reduce the operating temperature for energy saving. This work demonstrates a novel achievement of a response percentage of 273% at 100 ppm of H_2_S operating at RT. The evaluation of other parameters shows that the detection limit is 15 ppm, which is far below the dangerous concentration (100 ppm) of this harmful gas in air. Furthermore, the sensor exhibited a fast response time of 39 s, and excellent stability and selectivity toward H_2_S gas. As the sensor operates at RT and requires a low bias voltage of 0.5 V, the operation and production costs of the sensor are drastically reduced.

## 2. Materials and Methods

### 2.1. Materials

All chemicals, including cobalt nitrate hexahydrate (Co(NO_3_)_2_ 6H_2_O) and 2-methylimidazole (Hmim), were purchased from Sigma-Aldrich, U.S.A and used as received. Chitosan (Mw = 50,000–190,000 Da) and acetic acid were purchased from Polysciences, Warrington, PA, U.S.A. Glycerol ionic liquid (IL) was purchased from Quarek Corp, London, UK.

### 2.2. Synthesis of the ZIF-67 Powder

In a typical synthesis, ZIF-67 was prepared following a previously reported procedure [49]. Here, 0.45 g of (Co(NO_3_)_2_ 6H_2_O) was first dissolved in 3 mL of deionized (DI) water. Another 5.5 g of 2-mythelimidazole (Hmim) was dissolved separately in 20 mL of DI water. The metal solution was then added dropwise to the linker solution after both were dissolved completely. The mixed solution was stirred for 24 h at RT. The resulting purple product was then isolated through centrifugation, and it was washed thrice with DI water and methanol subsequently. The sample was then activated in a vacuum oven at 80 °C for 24 h.

### 2.3. Synthesis of the Membranes

**Figure 1:**
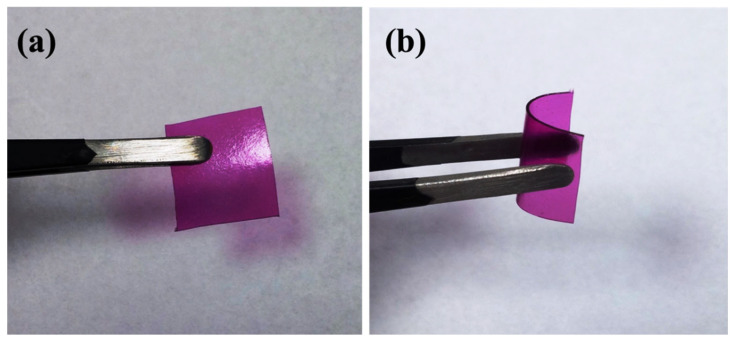
(**a**) A 1 cm × 1 cm piece of the fabricated membrane, (**b**) demonstration of the flexibility of the membrane.

The synthesized ZIF-67 was dispersed by varying wt% values in distilled water (DW) using a vortex shaker, and acetic acid was added to make a 3% solution. Then, 0.8 gms of CS was added along with 2 mL of glycerol IL. The synthesized 40 mL solution was kept under continuous stirring at 1450 RPM at RT for 24 h. The solution was then transferred to a petri dish and place in an oven at 70 °C for 18 h. The resultant membrane was subjected to various characterizations discussed in previous sections. CS–IL was doped with 2, 4, 5, and 6 wt% of ZIF-67 and fabricated following the above-mentioned protocols. The thickness of the membrane was measured and is tabulated in Table 1. Figure 1 shows a 1 × 1 piece of the membrane, demonstrating its flexibility.

### 2.4. Characterization

A Rigaku MiniFlex benchtop X-ray diffractometer with a CuKα radiation tube (λ = 1.542 Å) running at 40 kV over the range from 2–60° (2θ) and a rate of 2 °C min^−1^ was used to record the powder X-ray diffraction (PXRD) of ZIF-67. The surface morphology of the activated sample and its elemental analysis were analyzed using the scanning electron microscopy instrument the Quattro ESEM equipped with an energy-dispersive X-ray (EDX) detector operating at a high vacuum and a 30 kV accelerating voltage. Under a nitrogen atmosphere, the thermogravimetric analysis (TGA) of ZIF-67- and the ZIF-doped CS–IL membranes were obtained using a Mettler Toledo TGA2 analyzer, where the sample was kept in an aluminum pan adjusted to a heating rate of 10 °C min^−1^ and a heat range from 25 °C to 600 °C. The surface area and porosity were evaluated using N_2_ adsorption experiments, where the amount of gas adsorbed (cm^2^ g^−1^) was identified by the N_2_ adsorption–desorption isotherm as a function of relative pressure (P/P_0_). P is the N_2_ equilibrium pressure, and P_0_ is the saturated vapor pressure at 77 K. The sample was further activated under vacuum for 4 h and heated at 100 °C before the measurements.

### 2.5. Sensor Fabrication and H_2_S Gas Sensing Test

The sensing membranes (as 1 cm^2^ square pieces) were fabricated into sensor prototypes by placing them between a copper sheet serving as a bottom electrode and a stainless steel mesh serving as a top electrode [10,11]. The configuration was fastened using Kapton tape. The device was placed inside the gas chamber, and the electrical probes were then connected. The chamber was sealed to prevent any gas leakage and to maintain the humidity inside close to 0%. The gas testing sequences were executed using the LabVIEW software, which served as the interface between the computer and Keithley Instruments source measurement unit (KI 236). Mass flow controllers were deployed to expose the device to controlled amounts of test and flush gas. All through the testing sequences, the device was provided with a base voltage of 0.5 V and maintained at RT.

## 3. Results

### 3.1. Characterization of ZIF-67 Powder

**Figure 2:**
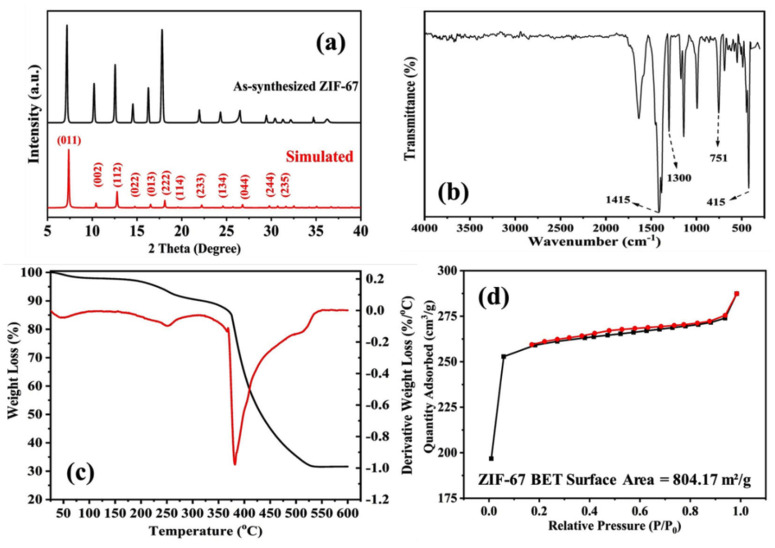
(**a**) Comparison of the X-ray diffraction patterns, (**b**) Fourier transform infrared spectra of the as-synthesized ZIF–67 powder, (**c**) thermogravimetric analysis curves, and (**d**) Brunauer–Emmett–Teller surface area analysis of the ZIF–67 powder.

The phase structure and purity of the prepared ZIF-67 sample were evaluated using powder X-ray diffraction (PXRD) patterns, which were compared with simulated ones (Figure 2a). The obtained diffraction patterns were in good agreement with the simulated patterns, confirming the successful synthesis of ZIF-67, which exhibits a cubic crystal system with unit cell parameters of a = b = c = 16.9589 Å [43,47].

The Fourier transform infrared (FTIR) analysis of the ZIF-67 powder (Figure 2b) shows a slight shift in the bands in comparison with those in the reported literature [15,16,17,50]. The spectra show a distinctive peak for the ZIF-67 at 415 cm^−1^ [17], which denotes the Co–N bond stretching vibration. The band at 751 cm^−1^ is assigned to the C=N stretching vibration, whereas the band at 1300 cm^−1^ corresponds to C=C stretching [15,38]. The band at 1415 cm^−1^ is attributed to CH_3_ bending vibration [39].

The thermal analysis of the ZIF-67 powder was conducted between RT and 600 °C (Figure 2c). Two weight drops were observed in the as-synthesized powder. The first drop of approximately 10% in weight loss was attributed to water molecules evaporating from the framework pores. A drastic decrease in weight was then observed between 390 °C and 540 °C (60% weight loss), indicating the total decomposition of the organic linker from the material. The remaining 40% weight was related to the metal oxide formed upon increasing the temperature.

Nitrogen sorption measurements were conducted to confirm the micropore characteristics of ZIF-67. As shown in Figure 2d, the isotherm loop indicated a micropore ZIF-67 material following a type I isotherm with a Brunauer–Emmett–Teller surface area of 804.17 m^2^/g and a maximum pore volume of 0.391 cm^3^/g, which is comparative to those reported in the literature [23,42,44,49]. The sudden uptake at a relative pressure of approximately 0.4 could be related to the physisorbed liquid nitrogen on the material surface of the nanoparticles [49,51].

**Figure 3:**
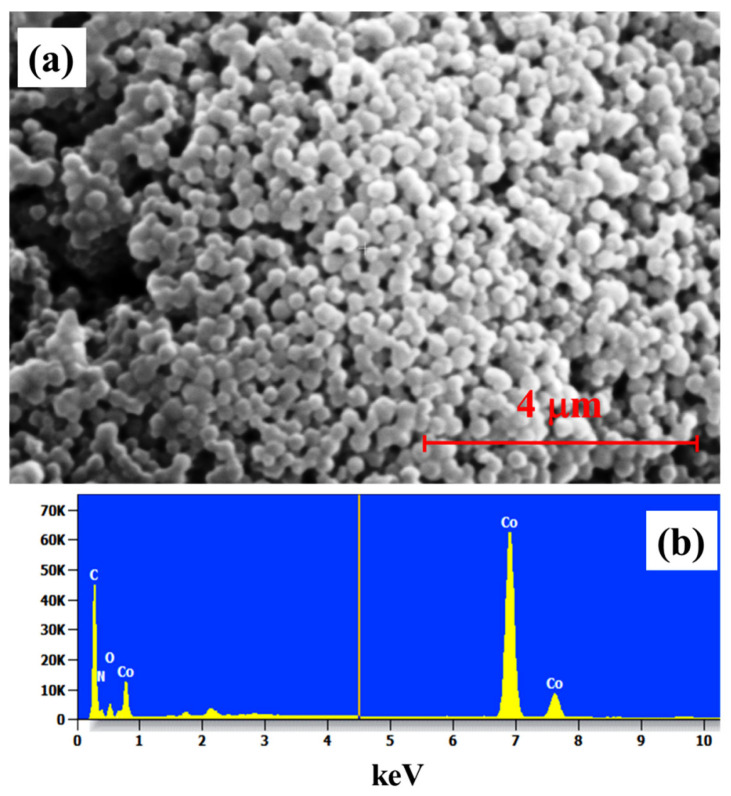
(**a**) Scanning electron microscopy images of ZIF-67 obtained at 4 µm, (**b**) energy-dispersive X-ray spectra of the ZIF-67 powder.

The morphological characterization (Figure 3a) of the as-prepared ZIF-67 shows agglomerates as nanoparticles, similar to those in previous reports [23,36,42,44,49]. Moreover, the presence of each expected element in the structure can be confirmed using energy-dispersive X-ray (EDX) analysis (Figure 3b).

### 3.2. Characterization of the ZIF-67-Doped CS–IL Membrane

**Figure 4:**
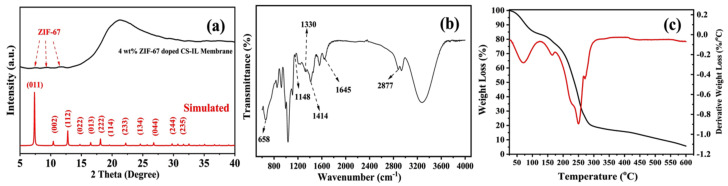
(**a**) X-ray diffraction pattern, (**b**) Fourier transform infrared spectra, and (**c**) thermogravimetric analysis curves of the ZIF–67-doped CS–IL membrane.

The XRD pattern of the 4 wt% ZIF-67-doped membrane (Figure 4a) shows a broad peak of the amorphous CS material that matches those of the reported literature [52]. Moreover, weak peaks of ZIF-67 particles were also made out as they were deeply embedded into the membrane. Figure 4b shows the FTIR analysis of the doped membrane. The spectra show vibrational modes of C–H bending at 658 cm^−1^, C–O stretching at 1158 cm^−1^ and 1654 cm^−1^, CH_3_ bending at 1414 cm^−1^ [38], a dimer OH at 2877 cm^−1^, and a C=C stretching mode at 1330 cm^−1^ [15,19,39,52]. The membrane showed relative peaks of ZIF-67 and CS [52], denoting the incorporation of ZIF-67 into the matrix.

Figure 4c shows the thermogravimetric analysis (TGA) of the doped membrane, recording a weight loss at approximately 100 °C attributed to the loss of adsorbed water molecules. The second loss in weight at up to 300 °C can be imputed to the decomposition of the organic groups in the membrane. There is a gradual loss in weight beyond 390 °C, which suggests the decomposition of the remainder of the linkers.

The CS–IL membranes were doped with different wt% values of ZIF-67 and subjected to 100 ppm of H_2_S gas to evaluate the optimum doping percentage. Table 1 records the sensitivity comparison of the membranes, and 4 wt% doping produced the best result. The thickness of the membranes was also measured using a screw gauge and are as tabulated below.

**Figure 5:**
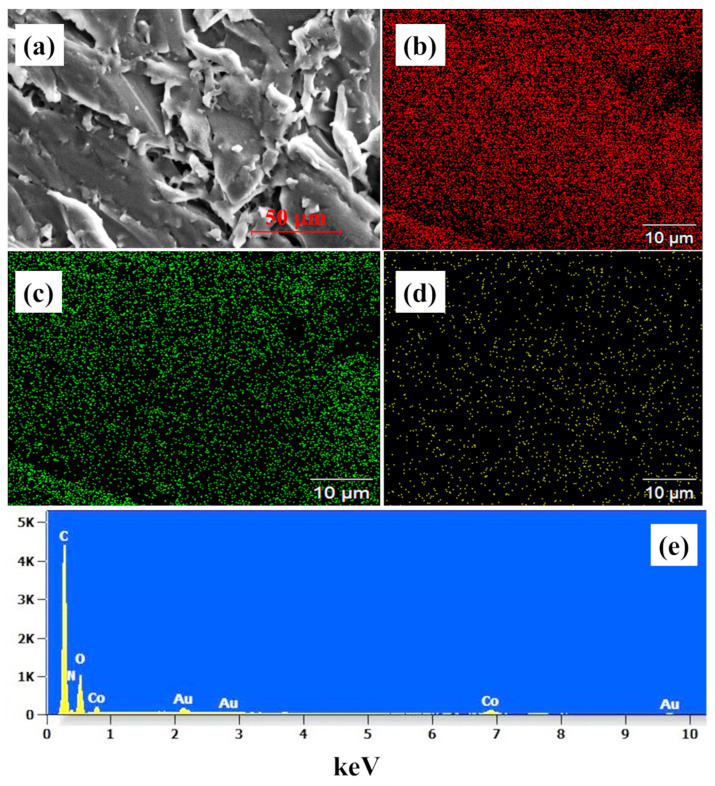
(**a**) SEM image of the ZIF-67-doped CS–IL membrane, (**b**–**d**) elemental mapping of the membrane showing carbon, oxygen, and cobalt, respectively, (**e**) EDX spectra of the membrane.

Initial SEM analysis showed dark spots embedded in the membrane, which was suspected to be ZIF particles. The surface of the membrane was mechanically etched using SiC sandpaper to ascertain this attribution. Figure 5a shows the SEM analysis of etched 4 wt% ZIF-67-doped CS–IL membrane. The elemental analysis of the etched membrane showed the presence of ZIF-67 particles (Figure 5e), and the mapping of the membrane ascertained the presence of the ZIF (Figure 5b–d).

### 3.3. Gas Sensing Performance

The sensor prototype was fabricated as detailed in our previous reports [10,11]. The sensor response toward H_2_S gas among other test gases was evaluated. Initially, CS doped with varying wt% of ZIF-67 was exposed to varying concentrations of H_2_S gas and synthetic air to evaluate the response in the presence and absence of the test gas. The sensor response was evaluated using Equation (1):

(1) $$S\;\left(\%\right)=\frac{R_{g}-R_{a}}{R_{a}}\times 100=\frac{\Delta R}{R_{a}}\times 100$$

where *R_a_* is the resistance of the sensor in synthetic air and *R_g_* is the resistance in the presence of the test gas. The sensor showed a very good response toward H_2_S gas. In this regard, Hani et al. reported that CS along with IL recorded a sensitivity of 200% toward 100 ppm of H_2_S and the lowest detection limit of 15 ppm with the operating temperature of 80 °C [26]. In this work, membranes were doped with 2, 4, 5, and 6 wt% of ZIF-67, which showed sensitivity toward H_2_S gas. The 4 wt% doping showed the best response toward the test gas at RT. The response values toward 100 ppm of H_2_S were 143% (2 wt%), 273% (4 wt%), 80% (5 wt%), and 7% (6 wt%) at RT, as detailed in Table 1. As the 4 wt% doping exhibited the best response, this membrane was further investigated in terms of the other aspects of the sensor’s parameters.

**Figure 6:**
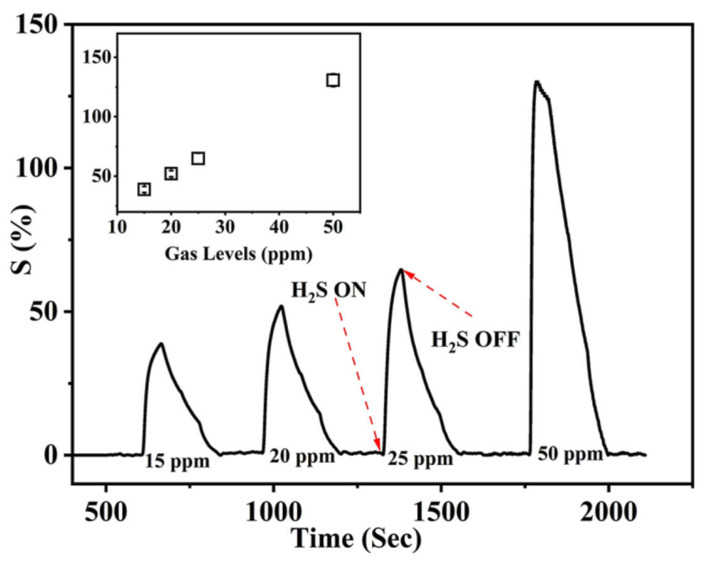
The sensitivity response of the sensor showing a lower limit of 15 ppm of H_2_S gas. The inset shows the response values of the membrane toward varying H_2_S concentrations.

The sensitivity aspect showed that the membrane showed a lower response of 15 ppm at RT (Figure 6). The inset shows the sensitivity values with respect to the gas concentration. Further tests were conducted at 100 ppm to evaluate the response toward the other test gases.

**Figure 7:**
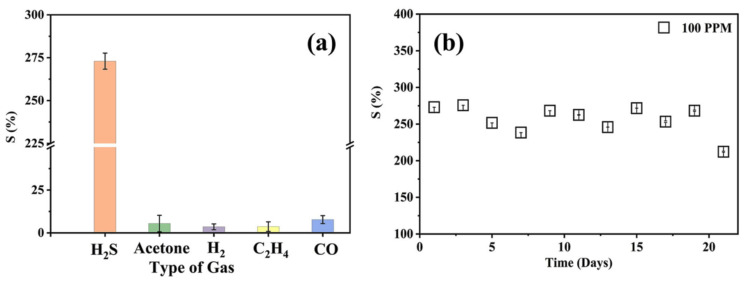
(**a**) Selectivity of the ZIF-67-doped CS-IL membrane sensor towards 100 ppm of different gases, (**b**) long-term stability of the membrane for 21 days.

The selectivity parameter evaluated that the sensor is highly selective towards H_2_S gas, which can be attributed to the inclusion of ZIF-67 crystallites. ZIF-67 provides additional basic sites for the adsorption of the acidic H_2_S protons via extended H-bonding formation, which is elaborated in the upcoming gas sensing mechanism section. The membrane also showed good repeatability and long-term stability when subjected to tests over 21 days (Figure 7b). Long-term stability tests show that the response of the sensor decreases slightly but always recovers when left undisturbed for 48 h. Nonetheless, the response of the sensor was very high compared with that of the previous study by Hani et al. [26], which had CS–IL as a standalone membrane detecting H_2_S at an operating temperature of 80 °C.

**Figure 8:**
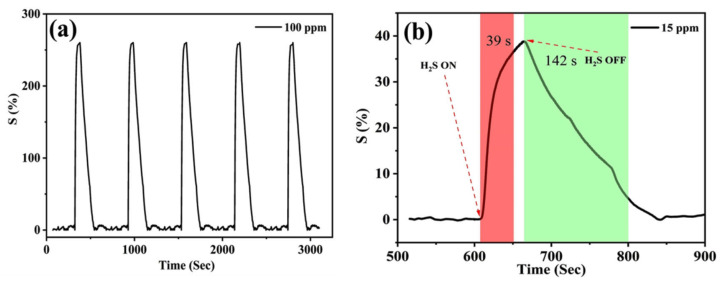
(**a**) Repeatability of the sensor at 100 ppm of H_2_S, (**b**) response (red) and recovery (green) time calculated for 15 ppm of H_2_S gas at RT.

The repeatability and stability aspects of the tests were conducted with exposure to five cycles of 100 ppm of H_2_S gas with the flushing of synthetic air in between each cycle to remove any residual gas molecules. The recorded response was 260.84 ± 0.54%, determining the repeatability aspect of the sensor as seen in Figure 8a. Because the lower response of the sensor was 15 ppm of H_2_S gas, the response and recovery time were also calculated from the data and were determined to be 39 s and 142 s, respectively (Figure 8b). The response time is defined as the time required for the sensor to attain 90% of its maximum response, whereas the recovery time is the time required for the sensor to recover to 10% of its baseline resistance after the target gas is stopped. Table 2 summaries the comparison of the reported literature with the current work to elucidate the performance of the sensor. Room temperature operation of the sensor with a low bias voltage makes it ideal for infield deployment as the operational and manufacturing costs are greatly reduced.

### 3.4. Gas Sensing Mechanism

The mechanism of the standalone CS–IL membrane was as outlined by Hani et al. [26]. The basic -NH_2_ groups along the chitosan chains provide sites of interaction with the protons of the acidic H_2_S gas molecules through H-bonding. This is further augmented with the presence of the highly hydroxylated ionic liquid. This interaction results in the enhanced charge transfer across the membrane, hence, improved gas sensitivity.

**Figure 9:**
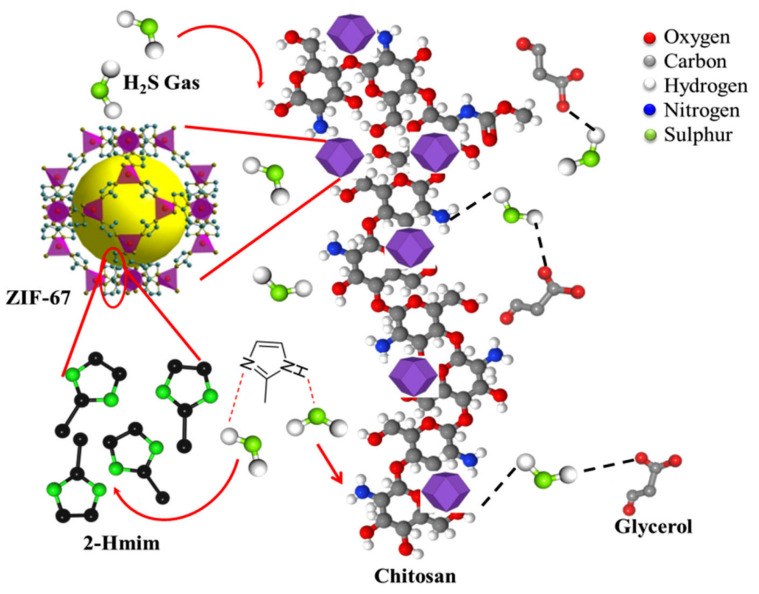
Sensing mechanism of the ZIF-67-doped CS–IL membrane.

In the current study, the chitosan matrix containing IL was further improved via the inclusion of ZIF-67 crystallites. The presence of N atoms present in the linker (2-Hmim) of the ZIF-67 provides additional basic sites for the adsorption of the acidic H_2_S protons via extended H-bonding formation. Therefore, the homogeneous distribution of the ZIF-67 crystallites within the chitosan matrix, as schematically presented in Figure 9, explains the enhanced sensitivity of the ZIF-67-doped chitosan membrane. In comparison to the report by Hani et al. [26], the inclusion of ZIF-67 crystallites enhanced the sensitivity of the matrix towards 100 ppm of H_2_S gas from 200% at 80 °C to 273% at RT. Our results show a consistent improvement in gas sensing with an increase in the proportion of ZIF-67 in the composite membrane up to 4 wt%. Upon increasing the concentration of ZIF-67 in the composite above 4 wt%, a decrease in gas sensitivity was observed, which could be related to the intrinsic resistance of the ZIF-67, whose effect dominated the behavior of the composite membrane, leading to the observed decline in the gas sensitivity of the membrane towards the target gas.

## 4. Conclusions

In this work, a flexible membrane from ZIF-67 mixed with the CS–IL solution was prepared for use as a highly sensitive and low-power-consumption gas sensor for environmental applications. ZIF-67 doped into the CS–IL matrix enhanced the sensing of H_2_S gas among other analytes. The addition of ZIF-67 into the organic matrix provided additional O–H groups, which subsequently enhanced the sensitivity response of the prototype with RT being considered as the operational temperature. The new sensor detected H_2_S gas at as low as 15 ppm at RT with a heightened response. The sensor demonstrated a good response, with response and recovery times of 39 and 142 s, respectively. The other aspects of the sensor, such as its long-term stability, repeatability, and selectivity, proved the enhanced performance of the material when compared with previously reported materials. Moreover, as the sensor works at RT and requires a low bias voltage of 0.5 V, the operational and production costs were significantly reduced for energy saving. The composite membrane is also known for its ecofriendly nature and can be commissioned as a real-time detection device for practical field applications.

## Figures and Tables

**Table 1:** Comparison of sensing for different doping percentages at 100 ppm of H_2_S gas.

Doping wt% (x)	Thickness	Sensing
CS + IL + (x)ZIF-67	μm	Response
		S%
2	268 ± 3	143 ± 1
4	262 ± 6	273 ± 6
5	270 ± 5	80 ± 2
6	283 ± 5	7 ± 2

**Table 2:** Sensor performance comparison with reported values in the literature.

Sensor/Material	Derivatives	Target Gas	Optimum Operating Temperature (°C)	Detection Limit (ppm)	Ref.
CS/IL	-	H_2_S	80	15	[26]
ZIF-67	Au/Co_3_O_4_	Acetone	220	100	[53]
	Co_3_O_4_/FGH		250	50	[31]
	Co_3_O_4_	Ethanol	300	200	[32]
	Co_3_O_4_		200	100	[54]
	-	Formaldehyde	150	100	[30]
	Co_3_O_4_	n-Butanol	100	21	[55]
ZIF-67/ZIF-8	-	H_2_	180	90	[20]
	ZnO/Co_3_O_4_	Acetone	275	1	[56]
ZIF-67/Ni-Co	Co_3_O_4_/NiCo_2_O_4_	H_2_S	250	50	[34]
ZIF-67	Co_3_O_4_	CO	180	90	[35]
SnO_2_/ZIF-67	-	CO_2_	205	5000	[41]
CS/IL/ZIF-67	-	H_2_S	23	15	THIS WORK

## Data Availability

Not Applicable.
